# Collectanea

**Published:** 1845-06

**Authors:** 


### Collectanea.

*Scarification of the Gums—Reasons for it.*—Dr. Marshall Hall says, that there is no practical fact, of the truth and value of which he is more satisfied than that of the effect and efficacy of scarification of the gums in infants, and not in infants only, but in children. "The process of teething," he observes, "is one of augmented arterial action and of vascular action generally; but it is also one of augmented nervous action; for formation, like nutrition, secretion, &c., generally, is always one of nervi-vascular action, and of this the case in question is, from its peculiar rapidity, one of the most energetic. Like other physiological processes, it is apt to become, from that very character of energy, pathological, or of morbid activity. It is obviously, then, attended with extreme suffering to the little patient; the brain is irritable, and the child is restless and cross; the gums are tumid and heated; there is fever, an affection of the general vascular system, and there are, too frequently, convulsions of various degrees and kinds, manifested in the muscles which move the eyeball, the thumb and finger, the toes; the larnyx, the parietes of the respiratory cavities; and the limbs and frame in general, affections of the excito-motor part of the nervous system, and of the secretions of the liver, kidneys and intestines, affections of the ganglionic division of that system.

"What is the precise cause and source of these formidable effects? Can the mere tension and irritation of the gum situated over the more prominent part of the teeth be the cause of such extensive morbid actions? I think not. The real source of these phenomona is in the entire dental system, in which actions of unusual energy and extent are going on—sub-inflammatory they might be called, were they not, in reality, of an essentially different nature and origin. This undue action takes place in the fangs and sockets of the teeth in their whole extent, with their connections, vascular, nervous, and membranous. But *the* focus from which the *nervous* actions emanate is, I believe, not as is generally imagined, the nerves of the mere *gums* seated over the prominent parts of the teeth, but the nerves which may be emphatically termed *the nerves of the teeth themselves,* the nerves which enter into the very fangs and substance of the teeth.

"*It is to the base of the gums, not to their apex merely, that the scarification should be applied.* The most marked case in which I have observed the instant good effect of scarification was one in which *all the teeth had pierced the gums!*

"This view of the subject may assist in removing the futile objections of some, who have, without due consideration, I am convinced, opposed my plan of frequent, often daily, scarification of the gums, to whom I would say, as my sole reply: Better scarify the gums *unnecessarily* one hundred times than allow the accession of one fit or convulsion from the neglect of this operation, which is equally important in its results and trifling in its character.

"And it is not merely the prominent and tense gum over the edges of the teeth which should be divided; the gums, or rather the blood-vessels, immediately over the very *nerves of the teeth,* should be scarified and divided, as you would divide the vessels of the conjunctiva in inflammation of that membrane.

"Now whilst there is fever or restlessness, or tendency to spasm or convulsion, this *local blood-letting* should be repeated daily, and in urgent cases even twice a day. I would here repeat my maxim: Better do this one hundred times unnecessarily than have one single fit from the neglect of so trifling an operation. A skilful person does it in a minute, and in a minute often prevents a most serious attack—an attack which may cripple the mind or limbs, or even take the life of our little patient, if frequently repeated. There is, in fact, no comparison between the means and the end, the one so trifling, the other so momentous."—*London Lancet, May,* 1844.

*Artificial Incubation.*—Since the public exhibition in Boston of the *eccalobeon,* a gentleman, who has a love for philosophical pursuits, has been experimenting with the machine, and his experience warrants him in assuring us that its economical powers are vastly over-rated. He cannot calculate upon much more than five chickens in a hundred eggs submitted to the artificial process. This small number invariably leave the shell on the nineteenth day, instead of the twenty-first, according to the known law of incubation with domestic fowls. Ninety-five per cent. therefore, are a dead loss. The young chicks are well formed, but seem to lack physical ability to force themselves from the shell. If opened for them at that juncture, they begin to show faint signs of life, but die within a few minutes. He believes, therefore, that the eccalobeon does not answer the expectations of the purchaser; and never has, under the most favorable circumstances, yielded more than five or six per cent. Either some essential part of the artificial method is kept back from those who have bought the right of using it, or the fact of the general failure to compete with nature, has not been honestly acknowledged. If any of our comparative anatomists, or other scientific correspondents, can explain this matter, they are invited to do so.

[*Boston Med. & Surg. Jour.*

*Boston Society of Natural History.*—This association, principally made up of physicians, is achieving wonders. The anniversary meeting, on

Wednesday last, was honored with the presence of ex-president Adams and other gentlemen of distinction. Rev. Charles Brook gave an old discourse, which was about as good as a new one. Certainly it has lost nothing by age.—*Ib.*

*Case of united Fracture of five years and a half duration undulated.*—Mrs. H., aged 65, married, had a fall five and a half years ago last fall, down a pair of stairs, striking on the tibia of the left side lightly, but with more force on the knee. She complianed of no pain in the tibia, and had severe pain in the knee, which increased much in size, and confined her to the bed for some months, and afterwards was obliged to use crutches, which she has never relinquished. Several medical gentlemen had seen her, and particularly Dr. Sweet, the bone-setter, who manipulated on the knee and lower joints several times, and insisted that the bone was reduced, believing it to be a luxation of the knee. On the 8th of January, 1845, I was requested by a physician to visit her with him on account of the enlarged state of the knee joint. On inspection and a slight manipulation, I concluded that it was one of hydrops articuli. The limb was very much flexed, and she could not make any extension with it, the contraction was so rigid. My attention was only drawn to the knee, no other part of the extremity being suspected as being injured or diseased. She was placed under the tinct. iodine externally, and hydriodate potassæ internally. There was much improvement in the knee, so that on my third visit she could move the limb freely, and extend it out as far as the right limb; the effusion had all been absorbed, and during this visit, on carrying my hand down the limb to notice the effects of the tinct. iodine on the cuticle which had left it in a state of desquamation, I happened to feel a slight depression on the tibia, three in. from the internal malleolus, and with a slight rotation discovered a crepitus at that point, and which was satisfactorily corroborated by the use of the stethoscope. The fracture was transverse, and would thus account for there not being any retraction at the end of the tibia, and also for her being able to stand upon the limb occasionally. Friction has been instituted with a proper apparatus to produce irritation of the ends of the bones, so that it can in time be properly adjusted. Should this not succeed, either the seton or the excision of the ends of the bone will be put in requisition.—*N. Y. Jour. Med.*

*Nasal Calculi.*—Monsieur Blandin extracted from the inferior meatus of the nasal fossæ, a calculous production which is to be deposited in Dupuytren's Museum. This stone is similar in appearance to a mulberry calculus; it is very hard, and would seem to be composed of carbonate, phosphate, and oxalate of lime. M. Blandin, who has on other occasions removed similar productions, is of opinion that they are formed by hardened mucosities which eventually become petrified.—*Abeille Medicale.*—*New York Journ. Med.*

*Growing Potatoes by Galvanism.*—On the 2d July, Mr. Wm. Ross presented to the New York Farmers' Club, some potatoes measuring seven inches in circumference. He planted the seed potatoes in drills on the 6th May last, using only leaves for manure. Across three rows at one end he buried a sheet of copper 5 feet long and 14 inches wide, and at the other end, 200 feet distant, a sheet of zinc of like dimensions. The sheets were placed in an upright position, and were connected by a copper wire, thus making a galvanic battery, the moisture of the earth completing the circuit. On the 15th, some potatoes were taken from these rows, varying from one inch to one inch and a quarter in diameter. On the 2nd July, others were dug that measured 2½ inches in diameter. Some of the adjoining rows beyond the battery, were tried, but few of them had potatoes larger than marrow-fat peas—certainly none larger than a boy's marble. Mr. Ross had made other experiments with electricity from a common Leyden jar, and by applying the charges three times, he succeeded in producing cucumbers in the open ground, which measured five inches in length in thirty-seven days from the time of planting the seed.—*Mech. Mag.—Louisville Med. Jour.*

*Is the Negro subject to Hare-lip?*—This may seem to be a question of no importance, and yet we feel desirous to ascertain the truth in the premises. Of the thousands of negroes we have seen in slave and free states, not one of any complexion, had hare-lip. We have conversed with gentlemen who have resided years in slave states, and their observation coincides with our own. Here and elsewhere, the deformity forces itself upon us, very frequently, but it is exclusively confined to the whites. Is this disparity in the races general throughout the United States; and if so, is there a philosophical reason for the difference? We put this inquiry to the profession at large, and very respectfully solicit their attention to it.—*Western Lancet.*

*Perpetual Feast of a Budhist.*—Mr. Abeel, under date of May 20th, last, then at Amoy, in China, says that the hospital is unusually full. Among the patients was a devotee of Budha, who, to his other meritorious deeds, had commenced a perpetual fasting—which consists in *eating all kinds of vegetables*, to the exclusion of meats. Besides the reward that will accrue to his spirit in the next world, for this prodigious self-denial, he believed that such a regimen had a miraculous influence on the mind, by clarifying its perceptions and endowing the soul with supernatural knowledge!

Absurd as this theory appears in a Pagan, it does not differ essentially from the doctrine that has been industriously promulgated by a modern school of reformers in this country. It was one of the everlasting propositions of that master-piece of prolixity, S. Graham, the hero of kitchens, that a vegetable diet exalted the intellectual powers, and elevated man in the scale of moral being—much higher, certainly, in the estimation of his branfed disciples, than ordinary philosophers were willing to admit. Happily for journalists as well as the public, the question of what is the proper food of man, has long since been settled. Man is an omnivorous animal. A few obstinate believers in the refining value of sour krout, raw turnips, chestnut puddings, and bean soups, still adhere to the skirts of their Northampton expounder of the laws of life—only indulging themselves in such unnatural, death-dealing horribles as roast beef and turkeys, on particular occasions, when the usages of society oblige them.—*Boston Med. & Surg. Journal.*

*Excision of the Teeth.*—Dr. Smethurst narrates a case in the London Lancet, where excision of a tooth, a natural one being pivoted on the fang, was followed by violent inflammation and suppuration of the injured part, attended with œdema of the whole head and face, and of the interior of the mouth and tongue, so that the patient was in danger of suffocation. Intense febrile excitement was also present. Bleeding, calomel and acetate of morphia relieved the attack, and, after a time, the pivoted tooth could be removed, when a discharge of pus and blood took place, with considerable relief. Ultimately the health was restored, and the lost tooth made good with a real one fixed with gold wires, which did not occasion any inconvenience whatever.—*Medical Times.*

*Case of Poisoning by Hydrocyanic Acid.*—On the evening of the 23d of January, I was summoned to the aid of Mr. H——, a medical gentleman of Stratton, near Cirencester, who was reported to have poisoned himself. I found him lying on his back on the hearth-rug, his head supported by a folded shawl. His countenance was placid, and free from all contortions, his eyes closed, and the pupils not largely dilated; a fresh healthy color was on his cheeks. His limbs were quite supple, and his body warm. Life had been extinct about ten minutes. From the statement made to me in the room, and which afterwards appeared in evidence at the inquest, I learnt that he had returned home from a long round of visiting, much fatigued, and feeling a pain in his chest, took the bottle of acid from its place in the surgery, and went into the parlor adjoining, for the purpose of taking a minim dose to relieve it—a remedy he had more than once had recourse to before, for the same purpose. While there he was heard to stagger, and as the house-keeper rushed into the room, he fell, and an ounce phial, about half full of hydrocyanic acid, of Scheele's strength, corked, dropped from his hand. She rang the bell violently, and gave the alarm, and in five minutes his brother, who is a medical man, was on the spot. He was then breathing, and his pulse was distinctly perceptible at the wrist. Notwithstanding every means tried to counteract the effects of the poison, he expired in a few minutes without any scream, and quite tranquilly.

*Appearances twenty-two hours after Death.*—Weather very cold. The body was cold and rigid. All the depending parts, as the back, shoulders, bend of elbows, &c., were of a mottled purplish color. On opening the chest, the right lung presented a dark, dusky purple appearance, was not much collapsed, and contained air. On being cut into, a frothy, dirty-brown, semi-mucous fluid exuded, tinged with blood. There was no odor of prussic acid from it. In the cavity of the right pleura were about eight ounces of thin serum; the surface of the pleura was not marked by any evidence of inflammation. The left lung was of a pale color, exsanguine, contained but little air, and poured out only a whitish frothy mucus on being cut into; it was firmly adherent in its whole extent to the costal pleura of the same side, and, posteriorly, the adhesions were so strong as to defy my strength to separate them. The pericardium was natural; it contained, perhaps, a little more fluid than usual in its cavity. The heart was small, and firmly contracted, and the vessels on its surface distended with fluid blood. On cutting into it, about three ounces of dark-colored fluid blood trickled out, without the least appearance of coagulation having been attempted. It exhaled no smell of prussic acid. The parietes of the ventricles were a little thicker than usual. The liver was large and healthy. The spleen soft and easily broken down, resembling mulberry jam. The kidneys were firm, rather large, and slightly coagulated. The stomach contained about fifteen ounces of half-digested food, that gave out the peculiar smell of food undergoing digestion, with which also could be satisfactorily recognized the well-known odor of bitter almonds. The mucous coat of the stomach was healthy, and smelt strongly of prussic acid after the stomach had been emptied of its contents. The intestines were healthy. The brain and its coverings were healthy, but its vessels and its sinuses were filled with dark-colored fluid blood. It was quite free from any smell of prussic acid.

In this case, first, he had power to cork the bottle after having taken the poison; indicating its paralyzing effects on the sensorium not to have been instantaneous. Second, the placid state of his features, unmarked by any act of expiring. Third, there was no scream, but he died tranquilly and silently. Fourth, the congested state of the right lung might more reasonably be referred to the effects of chronic pneumonia than to the poison. Fifth, the blood was everywhere dark colored and fluid. Sixth, the odor of bitter almonds was satisfactorily recognized in the stomach, and nowhere else. Seventh, he lived nearly ten minutes after having taken the poison.—Mr. Pooley, *in London Medical Gazette.*

*Dosing and Drugging.*—An anonymous correspondent has sent from New Bedford, Mass., a communication that appeared in one of the public papers of that town, which criticises, pretty keenly, a public lecture recently delivered there by Dr. Wm. A. Alcott, on what the speaker was pleased to call *dosing and drugging*. Not doubting the assertion that the discourse was not without objections, we do not feel at liberty to transfer the article to the

Journal, since it is minus a responsible signer. It is not enough that a communication is signed a "believer in science." We covet the whole of a writer's name under some circumstances, to fall back upon in case of necessity. Dr. Alcott, with a head full of radicalism on physiological subjects, notwithstanding his acknowledged philanthropy and excellence of heart, would be very likely to indulge in no very sparing thrusts against the practice of medicine, if it did not tally precisely with his own peculiar views.—*Boston Med. & Surg. Jour.*

*Reproduction of Bone in Necrosis.*—At a meeting of the Medico Chirurgical Society, Mr. Syme exhibited some specimens to show that new bone was formed by the periosteum. In the first place, he produced specimens of the imperfect reproduction which occurs in cases where a portion of bone is removed by mechanical violence, as in the operation of trepanning the skull. The loss of substance here is not completely supplied but only lessened, by a scanty growth of new bone round the margin of the aperture, diminishing in thickness from the circumference towards the centre, where there is usually a portion of the space occupied merely by a ligamentous membrane. He further illustrated this, by showing the result of experiments on dogs, performed in the way that Sir A. Cooper suggested, by removing a part of the radius, while the ulna was left entire. Here, too, the vacuity is very imperfectly provided with a substitute, by a conical shaped process of new bone, from each extremity of the old one, extending from the cut surface of the breach, and tapering towards each other, so as to leave a deficiency, occupied by a ligamentous texture. He next showed a similar result from disease in the human subject, nearly the whole shaft of the tibia having died and been discharged without the formation of a successor; the appearanee presented being precisely like that of the dog's bone just alluded to, and the imperfection of the limb so great as to require amputation several years after cicatrization of the sores. He then contrasted this preparation with one of necrosis in its ordinary form, where the old bone lies surrounded by a new shell ready to take its place in the event of removal. It is obvious, that this effectual reproduction cannot proceed from the remaining old bone, since it should in that case never be wanting, as there is always part of the shaft left entire, while it had been found that the bone possesses in itself very limited power of reproduction. The ample investing shell in question, therefore, not being attributable to the injury of the bone that retains its vitality, must be referred :—1. To separation and thickening of a lamina separated from the bone previous to its death. 2. To the ossification of organizable substance effused from the bone, previous to its death. 3. To ossification of the periosteum. 4. To ossification of the neighboring textures, whatever they may be. The arguments in support of these explanations being very plausible, it has always been considered most desirable to ascertain the truth, by examining the process which usually takes place. But where patients require amputation for necrosis, or sink under it, the disease is almost always either so recent or so far advanced, that the source of ossification cannot be recognized with certainty. In the course of fifteen years' hospital practice, Mr. Syme has met with only three cases that afforded an opportunity of dissection at the instructive period of formation, i. e., from three to six weeks after the commencement of the disease. Simple inspection of the preparations so obtained seems sufficient to satisfy all eyes, not obscured by preconceived opinions, that the new bone was formed on the inner surface of the periosteum. The osseous substance was deposited in crusts of uniform thickness, which implied an equality of reproductive action on the surface. The subjacent dead bone was perfectly smooth, which it could not have been if a lamina, however thin, had been detached from it. The periosteum was distinctly traced out and preserved, except at those parts where it had been destroyed by the disease; and it would have constituted the solace or defective portions of the osseous shell. In the early stage of the process of the new bone was distinctly deposited in separate masses, which were plainly seen to be insulated from each other when surveyed between the eye and a light.

[*London Forceps.*

*Malignant Tumor of the Inferior Maxilla.*—Mr. Hodgson exhibited a portion of the lower jaw, which he had removed by operation, affected with malignant disease, which had commenced in the laminated structure of the bone, and not, as is usual in the medullary membrane. Mr. Freer, House-Surgeon to the General Hospital, supplied the following notes of the case :—

A. Ward, aged 50, of mixed temperament, and has always enjoyed tolerable health. In the commencement of October she first perceived a small excrescence between the canine and cuspidate teeth on the left side of the inferior maxilla, and she suffered occasionally lancinating pain, with bleeding; it rapidly increased in size, pressing the teeth forwards, and spread over the double teeth on the same side. Early in November she had three teeth extracted, the tumor speedily filling up the space. On the 14th of November the tumor extended from the level of the angle of the inferior maxilla to the symphysis: its level was considerably above that of the surrounding teeth, and it projected considerably into the mouth. The bone was not thickened, nor were the integuments or neighboring glands at all implicated in the disease.

The tumor was cleanly scooped out so as to leave the alveolar structure denuded; its texture was the same as the specimen produced. The diseased structure did not appear to again encroach until the 1st of December, when it was necessary to daily destroy the new growth with escharotics, and these became insufficient to destroy it by the middle of December. The tumor continuing to encroach, Mr. Hodgson removed the portion of the left inferior maxilla from the symphysis to the angle.

Mr. Hodgson said that it appeared to be a disease of the medullary membrane. It was quite scooped out, by which every particle of disease seemed to be removed. It soon, however, showed itself again, when it was destroyed by nitric acid. After this it very soon came on again, and at the end of the week it had very much increased, which gave the idea that the disease originated in the medullary membrane. It was thought advisable to remove the diseased portion of the bone, and consequently the operation was performed this morning. Mr. Hodgson then described the operation. With respect to the dangers of the operation, Mr. Hodgson said that it had been stated that there was much danger of the patient being suffocated from the retraction of the tongue, and its closing the glottis at the time of division of the muscles connecting it with the maxilla. He had operated three times, and had not been aware of any such occurrence, and he did not think it had been noticed by other operators. He should rather attribute any symptoms of suffocation to the blood having found its way into the larynx, a circumstance not at all uncommon in operations connected with the tongue and palate.—*Med. Examiner.*

*Fibrous Tumor of the Inferior Maxilla.*—Mr. Hodgson then presented another tumor, which he had removed this morning from the inner portion of the inferior maxilla of a woman; it was easily removed. It was composed of a hard fibrous substance, interspersed with numerous spiculæ of bone.

Mr. Hodgson said that he removed a similar disease from a woman some years since; the disease returned in a very sluggish manner in the condyle of the maxilla, which he had not thought necessary to disarticulate at the time of taking out the diseased portion, but had cut the bone through just below it. He regretted now that he had not disarticulated the condyle, for he left only a small portion of the bone behind. The case, however, was going on very well. Mr. Hodgson does not believe that this disease is malignant, as some cases have been cured by the application of iodine and mercury, and they generally got well if completely removed.—*ib.*

*Multiplication of Dentists.*—At the present rate of increase, the dentists will out-number the physicians. There are nine in one short street in Boston, and how many more there are from Roxbury to Winnisimmit Ferry, the Directory does not mention. They all assert, likewise, that the demand for dentistry is increasing. This must undoubtedly arise from the bad work of some of the craft. It takes one half of them to repair the poor operations performed by the rest. Their income, too, quite outvies the charges of the profession. High as their fees are, the public bear the burden without wincing which proves that a competent man may get his own price for any undertaking. Dental societies are correcting the empiricism of the profession a little, but not quite fast enongh. Persons who never knew the composition of an artificial tooth, open an office, puff themselves, and gather customers as successfully as some of the best-taught graduates of the Baltimore College. If the abuse of dentistry could be corrected, the increase of numbers would create no alarm.

*On the Frequent Spontaneous Cure of Pulmonary Consumption, and the Indications furnished by Pathology for its Rational Treatment.*—Dr. J. Hughes Bennett states, that of seventy-three bodies he has examined since last November, he found puckerings or concretions in the lungs in twenty-eight. They were combined with induration alone in twelve, with cretaceous or calcareous concretions in sixteen. They occurred in the right lung seven times, in the left lung twice, and in both lungs nineteen times. He thinks that these observations, conjoined with those of Roger and Boudet, serve to establish that the spontaneous cure of pulmonary tubercle occurs in the proportion of from one-third to one-half of all the individuals who die after the age of 40. Dr. Bennett observes, that as empirical means for accomplishing a cure have notoriously failed, perhaps a study of the method in which nature operates may be more successful. There seems no reason why cavities in the lungs should not heal with the same frequency as ulcerations or abscesses in other internal organs, if the further deposition of tubercle could be arrested. This is only to be accomplished by overcoming the pathological conditions on which the deposition of tubercle depends. These are—first, a morbid state of the blood, the result of imperfect nutrition; secondly, local inflammation, by means of which an unhealthy exudation is poured out that assumes the form of tubercular or scrofulous matter. The indications for treatment are—1st, To overcome the dyspepsia and acidity in the alimentary canal; 2nd, To furnish the material necessary for the formation of a healthy chyme; and 3rd, To combat the local inflammation. The dyspepsia and vomiting are often to be alleviated by naphtha. He attributes the good effects of this remedy to its power of allaying the irritability of the stomach, and thus enabling the patient to take nourishment. In following the second indication, he now, after four years' employment of it in private, as well as in dispensary and hospital practice, strongly recommends cod-liver oil as a most valuable remedy.—*Edinburgh Medical and Surgical Journal.*

*Prestat's Adhesive Plaster.*—The following composition is said never to crack, and not to inflame the skin:—Empl. diachyl. gum, 400 grains; purified rosin, 50 grains; tereb. venet., 38 grains, are mixed together at a gentle heat, and then 12 grains of gum mastich and 12 grains of gum ammoniac incorporated, and the mass spread on linen. In winter it is advisable to add 10 grains more turpentine, and 12 grs. ol. amygdal.—*Journ. für Prakt. Chem.—Louisville Med. Journ.*

